# β-amyloid monomers drive up neuronal aerobic glycolysis in response to energy stressors

**DOI:** 10.18632/aging.203330

**Published:** 2021-07-21

**Authors:** Rosa Santangelo, Maria Laura Giuffrida, Cristina Satriano, Marianna Flora Tomasello, Stefania Zimbone, Agata Copani

**Affiliations:** 1 Department of Drug and Health Sciences, University of Catania, Catania 95125, Italy; 2 Institute of Crystallography, National Council of Research, Catania Unit, Catania 95126, Italy; 3 Department of Chemical Sciences, University of Catania, Catania 95125, Italy

**Keywords:** Alzheimer’s disease, β-amyloid monomers, aerobic glycolysis, default mode network, lactate, oligomycin, kainate, AMPK

## Abstract

Research on cerebral glucose metabolism has shown that the aging brain experiences a fall of aerobic glycolysis, and that the age-related loss of aerobic glycolysis may accelerate Alzheimer’s disease pathology. In the healthy brain, aerobic glycolysis, namely the use of glucose outside oxidative phosphorylation, may cover energy demand and increase neuronal resilience to stressors at once. Currently, the drivers of aerobic glycolysis in neurons are unknown. We previously demonstrated that synthetic monomers of β-amyloid protein (Aβ) enhance glucose uptake in neurons, and that endogenous Aβ is required for depolarization-induced glucose uptake in cultured neurons. In this work, we show that cultured cortical neurons increased aerobic glycolysis in response to the inhibition of oxidative phosphorylation by oligomycin or to a kainate pulse. Such an increase was prevented by blocking the endogenous Aβ tone and re-established by the exogenous addition of synthetic Aβ monomers. The activity of mitochondria-bound hexokinase-1 appeared to be necessary for monomers-stimulated aerobic glycolysis during oxidative phosphorylation blockade or kainate excitation. Our data suggest that, through Aβ release, neurons coordinate glucose uptake with aerobic glycolysis in response to metabolic stressors. The implications of this new finding are that the age-related drop in aerobic glycolysis and the susceptibility to Alzheimer’s disease could be linked to factors interfering with release and functions of Aβ monomers.

## INTRODUCTION

Recent evidence shows that the aging human brain suffers a fall of aerobic glycolysis (AG) (i.e., the use of glucose outside oxidative phosphorylation, OP) rather than a global decrease in glucose metabolism [1]. AG accounts for about 10–12% of glucose used by the adult brain and has its highest levels in the default mode network (DMN) [2], where it markedly drops with aging [1].

The DMN is a cluster of regions underlying the ability of the young adult brain to maintain the self-referential functions, and showing hypoactivity and Aβ deposition in older adults with Alzheimer’s disease (AD) [3]. AG has been associated with synaptic activity [4] and is considered an adaptive advantage over OP for those areas requiring a fast ATP supply to active synapses and a fast synaptic turnover [1, 5]. The high intrinsic activity of the DMN, possibly permitted by AG, and the ensuing activity-driven Aβ release [6], could explain the topographical association between DMN, AG and Aβ deposition [2–3].

A recent study by Vlassenko and colleagues has shown that in individuals with significant Aβ load, lower AG is associated with higher tau deposition [7], suggesting that an age-related loss of AG may accelerate AD pathology. Accordingly, it has been hypothesized that, by diminishing mitochondrial activity and reactive oxygen species (ROS) production, AG could represent a pre-emptive protective mechanism against neuronal stressors [8].

The reason why brain AG falls with age, and particularly in the DMN, favoring the appearance of AD pathology [7] is not clear. Although astrocytes are the major site of AG in the brain [9–10], recent evidence shows that neurons undergo AG during activation [11–13]. We previously demonstrated that Aβ_42_, in the form of physiological monomer, is endowed with a broad neuroprotective activity [14], and enhances glucose uptake in neurons by activating type-1 insulin-like growth factor receptors (IGF-IRs) [15]. Moreover, we found that Aβ release is required for depolarization-induced glucose uptake in neurons, and that amyloid precursor protein (APP)-null neurons fail to enhance depolarization-stimulated glucose uptake unless exogenous Aβ_42_ monomers are added [15]. Thus, Aβ released at the synapses could be critical for maintaining neuronal glucose homeostasis.

In APP transgenic mice, prior to plaque deposition, neuronal activity appears to co-regulate the regional concentrations of interstitial fluid Aβ and lactate (the end-product of AG) [16], suggesting to us that secreted Aβ could drive AG beside promoting neuronal glucose uptake. Given our previous demonstration that Aβ_42_ monomers selectively activate IGF-IRs to enhance glucose uptake in neurons [15], in close similarity with the contribution offered by the IGF-1 signaling pathway to the high glycolytic flux of many tumor cells [17], it is conceivable that Aβ_42_ monomers may support neuronal AG via IGF-IR activation.

By using long term primary neuronal cultures, virtually devoid of potentially confounding glia cells, we aimed at exploring whether physiological forms of Aβ can promote AG in neurons under basal and/or metabolic stress conditions. The novel demonstration of a causal link between the endogenous Aβ tone and neuronal AG would suggest that the intrinsic vulnerability of the aging brain to different stressors (including β-amyloidosis) could stay in those factors that interfere with the release and functions of physiological Aβ monomers.

## RESULTS

### The endogenous release of Aβ sustained neuronal survival and AG when OP was inhibited

Experiments were carried out in primary cultures of pure cortical neurons, obtained from E15 rat embryos according to a well-established protocol that produces 99% neurons [18]. Neuronal cultures were grown for over two weeks by replacing half the volume of the culture media every 3 or 4 days. Mature neurons (14–17 DIV) were maintained for 2 hours (hr) in artificial excitable cerebrospinal fluid (CSF) (i.e., containing 10 μM glycine), lacking glucose, and then forced to uptake new glucose added at a known concentration (i.e., the 3 mM concentration that lies in the normal CSF glucose range). We have previously shown that in glucose-starved cultures, the addition of synthetic Aβ_42_ monomers, 100 nM for 15 minutes (min), stimulates neuronal glucose uptake through the activation of IGF-IRs [15].

**Figure 1:**
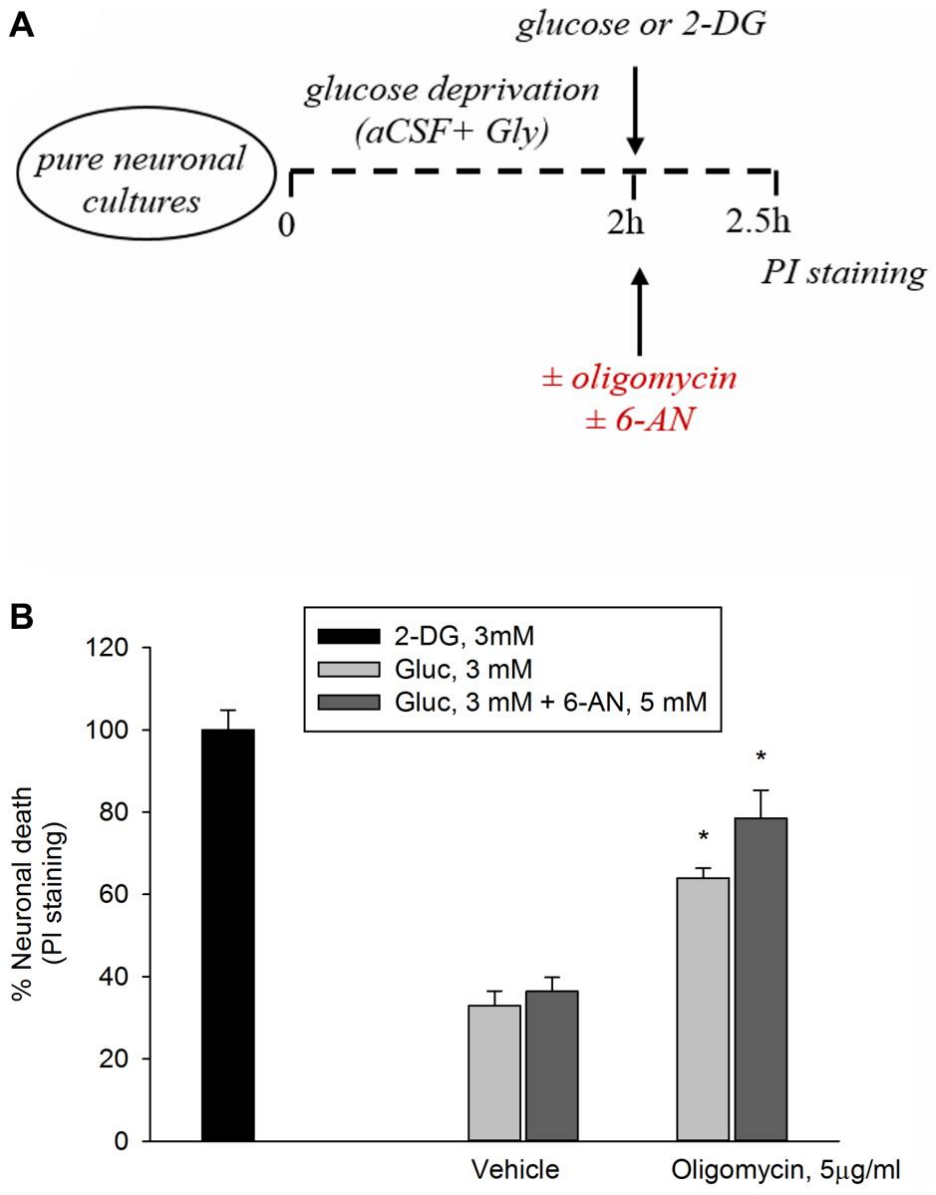
**Inhibition of the PP pathway by 6-aminonicotinamide did not increase significantly oligomycin toxicity.** Experiments were performed as represented in the drawing. (**A**) Following 2 hours of glucose deprivation, neurons were returned to 3 mM glucose (Gluc) in either the absence or the presence of oligomycin (5 μg/ml) to inhibit ATP synthase. (**B**) Oligomycin induced a 60% neuronal death after 2.5 hours, whereas 2-deoxyglucose (2-DG, 3 mM), which inhibits overall glucose metabolism, virtually killed all neurons. The addition of 6-aminonicotinamide (6-AN, 5 mM) did not potentiate significantly oligomycin toxicity. Neuronal death was quantified by propidium iodide (PI) staining of neurons that had lost membrane integrity and expressed as percentage of 2-DG-induced death. Bars represent the means ± SEM of 4 determinations. ^*^P < 0.001 vs. 2-DG; one-way ANOVA with post hoc Fisher LSD multiple comparison method.

In this set of experiments, following 2 hr of glucose deprivations, neurons were returned to 3 mM glucose in either the absence or the presence of oligomycin (5 μg/ml) to inhibit ATP synthase, with ensuing reduction of mitochondrial OP. Oligomycin induced a 60% neuronal death after 2.5 hr, whereas 2-deoxyglucose (2-DG, 3 mM), which inhibits overall glucose metabolism, virtually killed all neurons (Figure 1A, 1B). Inhibition of the pentose phosphate (PP) pathway (i.e., a metabolic pathway parallel to glycolysis) by 6-aminonicotinamide (6-AN, 5 mM) did not potentiate significantly oligomycin toxicity, suggesting that neuronal survival did not depend greatly on the PP pathway when OP was blockade (Figure 1A, 1B).

**Figure 2:**
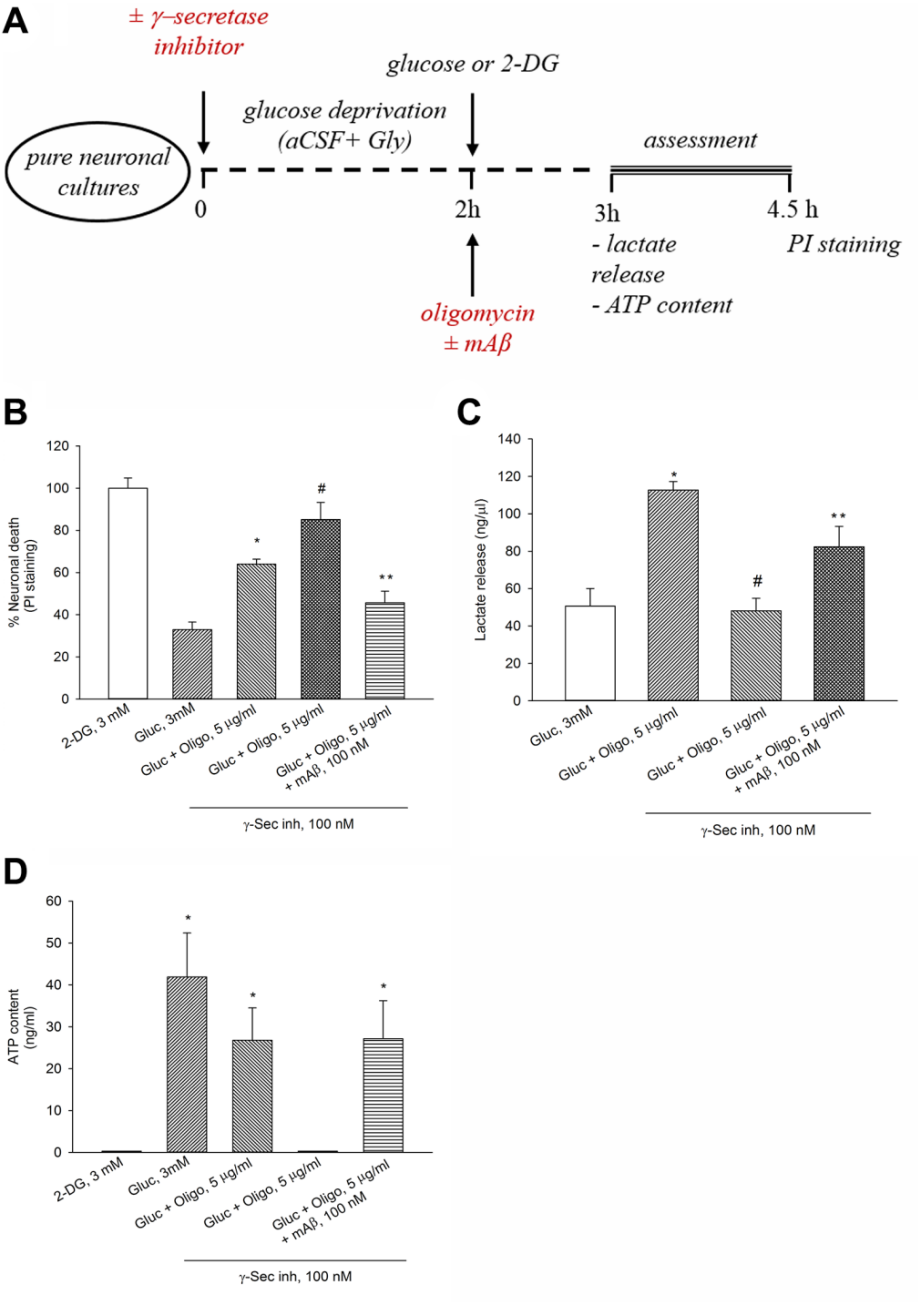
**The endogenous release of Aβ sustained neuronal survival, lactate release and ATP levels in the presence of oligomycin.** Experiments were performed as represented in the drawing (**A**). In the presence of oligomycin (Oligo, 5 μg/ml), blockade of Aβ production by γ-secretase inhibitor IX (γ-Sec Inh, 100 nM) worsened neuronal survival (**B**), reduced lactate release (**C**) and ATP content (**D**). The addition of synthetic Aβ_42_ monomers (mAβ, 100 nM) prevented the effects of γ-Sec Inh (**B**–**D**). Both in (**B** and **C**) bars represent the means ± SEM of 4 determinations. *P* < 0.001 vs. ^*^glucose (Gluc), or ^#^Gluc ± Oligo in the absence of γ-Sec Inh, or ^**^Gluc ± Oligo in the presence of γ-Sec Inh; one-way ANOVA with post hoc Fisher LSD multiple comparison method. In (**D**) bars represent the means ± SEM of 3–4 determinations. ^*^P < 0.05 vs. 2-DG or Gluc ± Oligo in the presence of γ-Sec Inh; one-way ANOVA with post hoc Fisher LSD multiple comparison method.

We have previously shown that γ-secretase inhibitor IX (100 nM for 2 hr) blocks the endogenous production of Aβ_42_ under basal conditions or following neuronal excitation [15]. Interestingly, when OP was impeded by oligomycin, blockade of Aβ_42_ production by γ-secretase inhibitor IX worsened neuronal survival, whereas the exogenous addition of synthetic Aβ_42_ monomers (at the known effective concentration of 100 nM [14]) opposed the effects of γ-secretase inhibitor IX (Figure 2A, 2B). Hence, endogenous Aβ was required to sustain neuronal survival following OP blockade. To investigate the relationship between neuronal survival under OP blockade and the trigger of AG (i.e., the glycolysis of glucose to lactate), we measured the lactate concentration in the bathing medium of neurons treated with oligomycin for 1 hr (Figure 2A, 2C). Basal lactate concentration varied widely across the different cultures, ranging from 10 ng to 200 ng/μl, depending on neuronal density and maturation age. Lactate concentration raised more than 2 folds in oligomycin-treated neurons with respect to the controls (Figure 2C). Noteworthy, a pre-treatment with γ-secretase inhibitor IX reduced lactate release in oligomycin-treated neurons, whereas the exogenous addition of synthetic Aβ_42_ monomers (100 nM) opposed the effects of γ-secretase inhibitor IX once again (Figure 2C). In the absence of oligomycin, neither blocking of Aβ production (up to 3 hours), nor adding synthetic Aβ_42_ monomers, which however stimulates glucose uptake [15], affected lactate release (ng/μl: basal = 50.63 ± 9.4; γ-secretase inhibitor IX = 44.15 ± 10.4; Aβ_42_ monomers = 47.97 ± 11.56; γ-secretase inhibitor IX + Aβ_42_ monomers = 48.82 ± 6.3). Under OP blockade, the total neuronal ATP content tended to decrease not significantly (Figure 2D), indicating that the production of ATP by forced AG largely compensated for the inhibition of mitochondrial ATP production. Following the pre-treatment with γ-secretase inhibitor IX, the ATP content fell below the detection limits (0.1 ng/ml) in oligomycin-treated neurons, whereas it increased again with the addition of synthetic Aβ_42_ monomers (Figure 2D). Hence, both lactate release (Figure 2C) and ATP levels (Figure 2D) appeared to depend on Aβ production following neuronal exposure to oligomycin. Interestingly, when Aβ release and OP were both blocked, the ATP levels dropped markedly with respect to the controls (Gluc 3 mM) (Figure 2D), whereas lactate levels were comparable between the two conditions (Gluc 3 mM vs. Gluc + Oligo in the presence of γ-Sec Inh) (Figure 2C). It follows that the ATP levels associated with basal lactate production (which did not rely on endogenous Aβ) were probably negligible. Overall, these data indicate that endogenous Aβ was required to sustain AG when OP was blockade.

### Blockade of endogenous Aβ prevented kainate-stimulated AG

**Figure 3:**
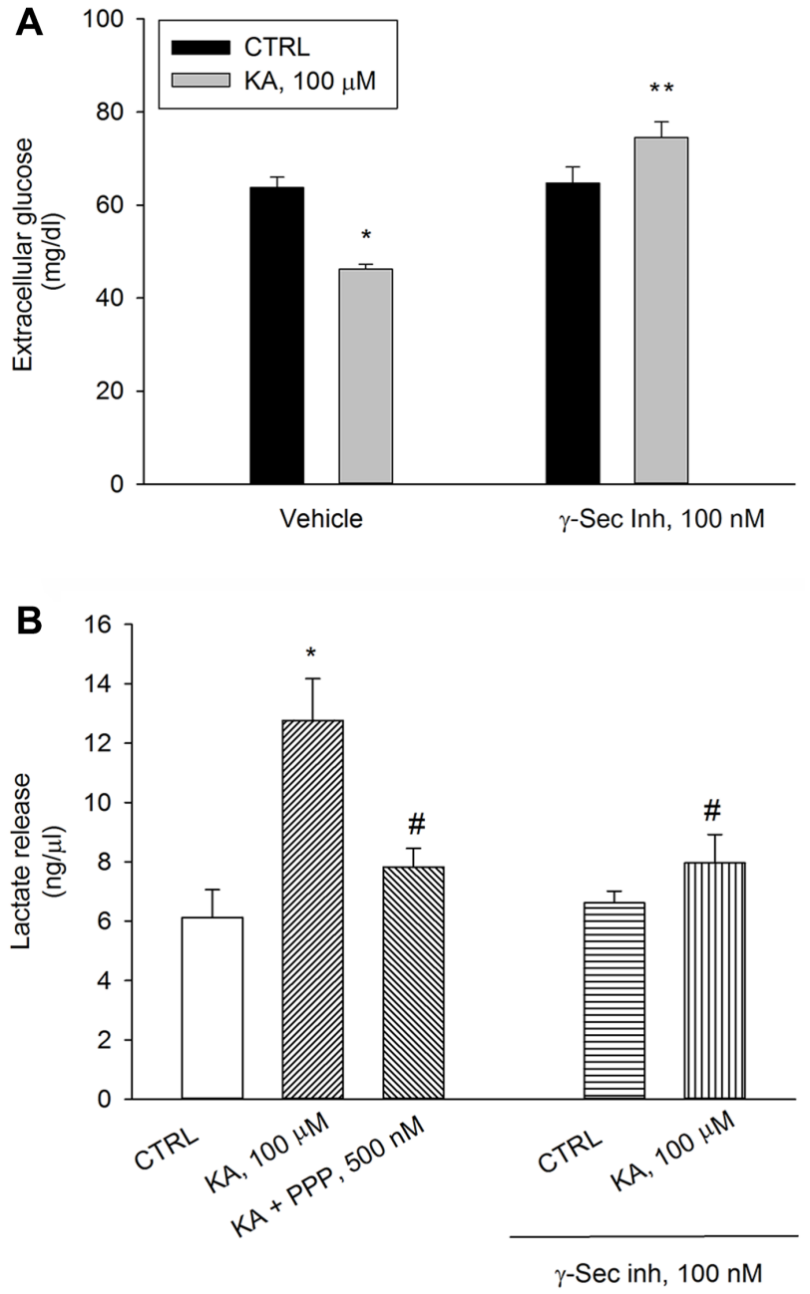
**Inhibition of Aβ release or blockade of IGF-IRs prevent kainate-stimulated lactate release.** Following 2 hours of glucose deprivation, 3 mM glucose was added to neuronal cultures. A treatment with kainate (KA, 100 μM) stimulated glucose uptake after 10 min (**A**) and lactate release after 40 min (**B**). Glucose consumption was measured as glucose (mg/dl) remaining in the incubation buffer 10 minutes following re-addition. With respect to the initial 3 mM glucose concentration, no glucose uptake occurred within 10 min unless KA was added. The IGF-IR antagonist, PPP (500 nM), and γ-secretase inhibitor IX (γ-Sec Inh, 100 nM) prevented kainate-stimulated lactate release at 40 min (**B**). Bars represent the means ± SEM of 4 determinations. In (**A**) *p* < 0.001 vs. ^*^control (CTRL) or ^**^KA alone. In (**B**) *p* < 0.001 vs. ^*^CTRL or ^#^KA alone; one-way ANOVA with post hoc Fisher LSD multiple comparison method.

We have previously shown that a depolarization pulse with KCl (40 mM for 15 min) evokes a significant increase in neuronal glucose uptake, which is prevented by either γ-secretase inhibitor IX or the IGF-IR antagonist, PPP, (i.e., by blocking Aβ production or Aβ activity) [15]. Similar to the depolarizing pulse with KCl, the application of kainic acid (100 μM for 10 min) promoted glucose uptake in neurons, which was prevented by the pre-exposure to γ-secretase inhibitor IX (100 nM) (Figure 3A). In parallel, at 40 min, lactate concentration raised in kainate-treated neurons and the rise was prevented by the pre-exposure to γ-secretase inhibitor IX. γ-Secretase inhibitor IX per se was ineffective (Figure 3B). PPP (500 nM) prevented kainate-stimulated lactate release to the same extent of γ-secretase inhibitor IX, (Figure 3B). Thus, the endogenous production of Aβ and the endogenous activation of IGF-IRs were required to sustain AG during neuronal activation.

### Inhibition of AMP-activated protein kinase (AMPK) did not prevent the up-regulation of AG due to Aβ monomers

**Figure 4:**
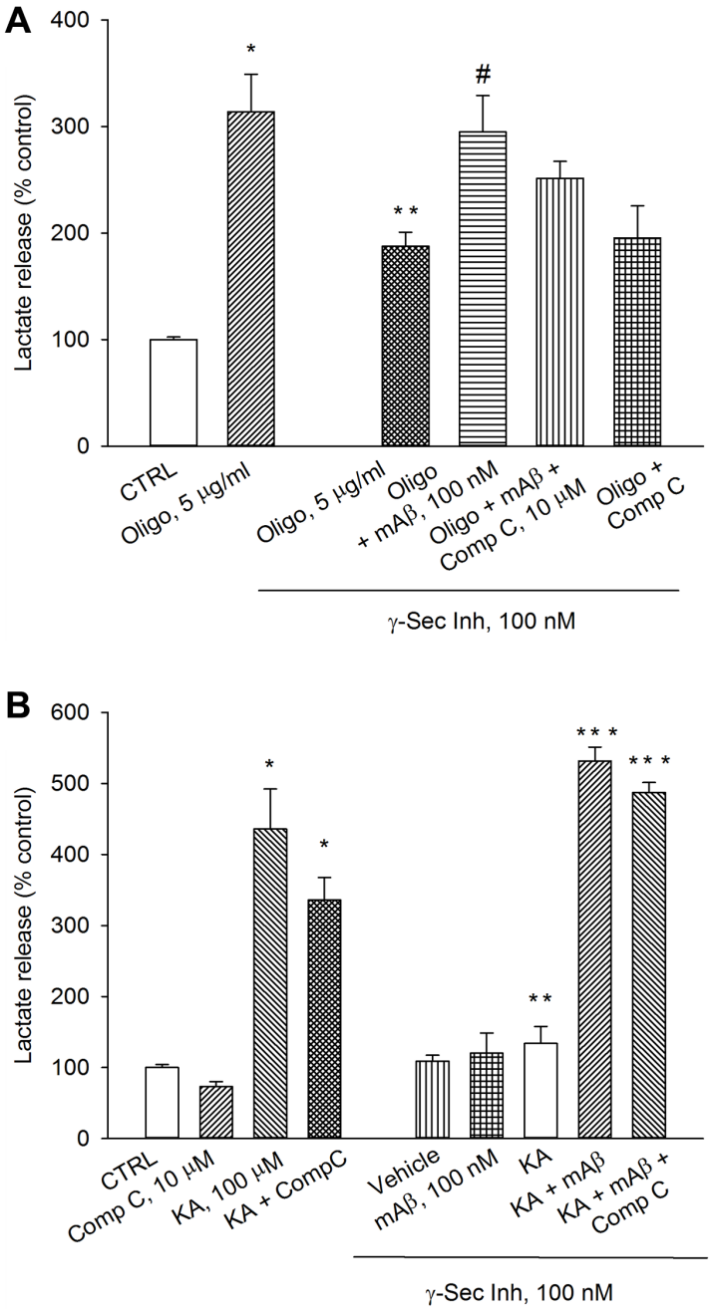
**Inhibition of AMPK by Compound C did not prevent lactate production due to Aβ release.** Neurons were glucose-starved for 2 hours before returning to 3 mM glucose. γ-Secretase inhibitor IX (γ-Sec Inh, 100 nM) reduced lactate release both in oligomycin-treated neurons (Oligo, 5 μg/ml for 1 hr) (**A**) and kainate-treated neurons (KA, 100 μM for 40 min) (**B**). The addition of synthetic Aβ_42_ monomers (mAβ, 100 nM) prevented the reduction of lactate release, induced by γ-Sec Inh, both in (**A** and **B**). Compound C (10 μM), did not affect significantly the rescuing effect of exogenous Aβ_42_ monomers in either (**A** or **B**). Bars represent the means ± SEM of 4 determinations. In (**A**) *p* < 0.001 vs. ^*^control (CTRL) or ^**^Oligo in the absence of γ-Sec Inh, and *p* < 0.05 vs. ^#^Oligo + γ-Sec Inh. In (**B**) *p* < 0.001 vs. ^*^control (CTRL) or ^**^KA in the absence of γ-Sec Inh, and *p* < 0.001 vs. ^***^KA + γ-Sec Inh; one-way ANOVA with post hoc Fisher LSD multiple comparison method.

The AMPK is able to reprogram cell metabolism for adaptation to energy stress [19]. Therefore, we investigated whether AMPK was required to sustain Aβ-mediated AG under conditions that interfere with ATP production (e.g., exposure to oligomycin) or accelerate ATP consumption (e.g., exposure to kainate). As before, a pre-treatment with γ-secretase inhibitor IX reduced lactate release in oligomycin-treated neurons, which was rescued by the exogenous addition of synthetic Aβ_42_ monomers (Figure 4A). The addition of the AMPK inhibitor, Compound C (10 μM), to exogenous Aβ_42_ monomers showed a trend toward the reduction of lactate release that, however, did not reach significance. Compound C per se was ineffective (Figure 4A). γ-Secretase inhibitor IX completely prevented kainate-induced lactate release, which was re-established by exogenous Aβ_42_ monomers (Figure 4B). Even in this case, the addition of the AMPK inhibitor, Compound C (10 μM), did not affect the rescuing effect of exogenous Aβ_42_ monomers (Figure 4B). In the absence of γ-secretase inhibitor IX, Compound C slightly reduced both basal and kainate-stimulated lactate release. Blockade of basal Aβ production and re-addition of Aβ_42_ monomers, in the absence of kainate, were ineffective (Figure 4B). Hence, endogenous Aβ was required to sustain AG in kainate-stimulated neurons. In addition, AMPK did not seem to be required to support Aβ-mediated AG under metabolic stresses (i.e., neuronal exposure to oligomycin or kainate).

### Mitochondria-bound HK-1 was needed for Aβ-mediated AG under metabolic stresses

We have previously shown that endogenously released Aβ, similarly to synthetic Aβ_42_ monomers, activates IGF-IRs to start glucose uptake in neurons [15]. Here, blockade of IGF-IRs by PPP, similar to the blockade of Aβ production, prevented kainate-stimulated lactate release (Figure 3B), suggesting that glucose uptake and lactate release lie along the same pathway activated by Aβ. The IGF-IR/Phosphatidylinositol 3-kinase (PI-3K)/AKT pathway can promote the binding of HK-1 to the outer mitochondrial membrane (OMM) [20], thus allowing the rapid formation of large amounts of glucose-6-phopshate (Gluc-6-P) and, consequently, of pyruvate that cannot be oxidized readily by the mitochondria and is diverged toward lactate production [20–21]. HK-1 is normally 75%–90% bound to mitochondria in neurons [22] and, therefore, small increments of mitochondria-bound HK-1 could be functionally significant.

**Figure 5:**
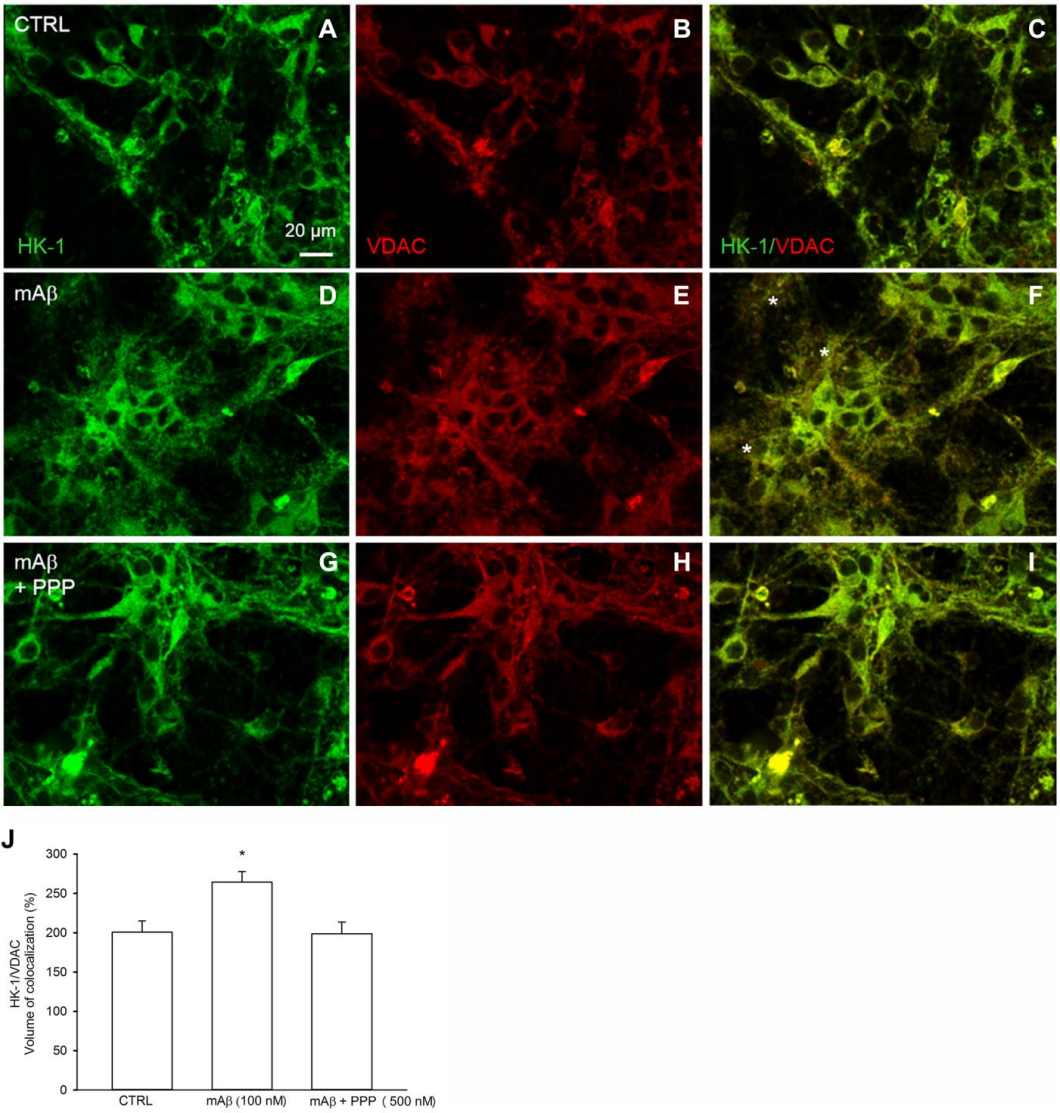
**Synthetic Aβ_42_ monomers increased the mitochondrial localization of HK-1 at the neurite processes in a manner dependent on IGF-IR activation.** Confocal images of primary cortical neurons glucose-starved for 2 hours before returning to 3 mM glucose, in the absence (CTRL, **A**–**C**) or in the presence of either 100 nM synthetic Aβ_42_ monomers for 40 min (mAβ, **D**–**F**) or synthetic Aβ_42_ monomers + 500 nM PPP (mAβ + PPP, **G**–**I**). Neurons were immunostained for HK-1 (green fluorescence) and VDAC (red fluorescence). Overlays of green and red fluorescence for each experimental conditions are shown on the right side of the figure (**C**, **F**, **I**). In (**F**) asterisks indicate neurite processes exhibiting green (HK-1)/red (VDAC) co-localization (orange to yellow). Images were not altered in any way, but were despeckled by ImageJ to reduce noise. Scale bar = 20 μm. In (**J**), bars represent the % image volume colocalized (i.e., the percentage of voxels which have both green (HK-1) and red (VDAC) fluorescence intensity above the threshold with respect to the total number of pixels in the image) for each experimental conditions, and values are expressed as means ± S.E.M. of 3 determinations. Each determination represented a culture dish in which the % of image volume colocalized was calculated from three random fields. ^*^p < 0.001 vs. control (CTRL); one-way ANOVA with post hoc Holm-Sidak multiple comparisons vs. control group.

**Figure 6:**
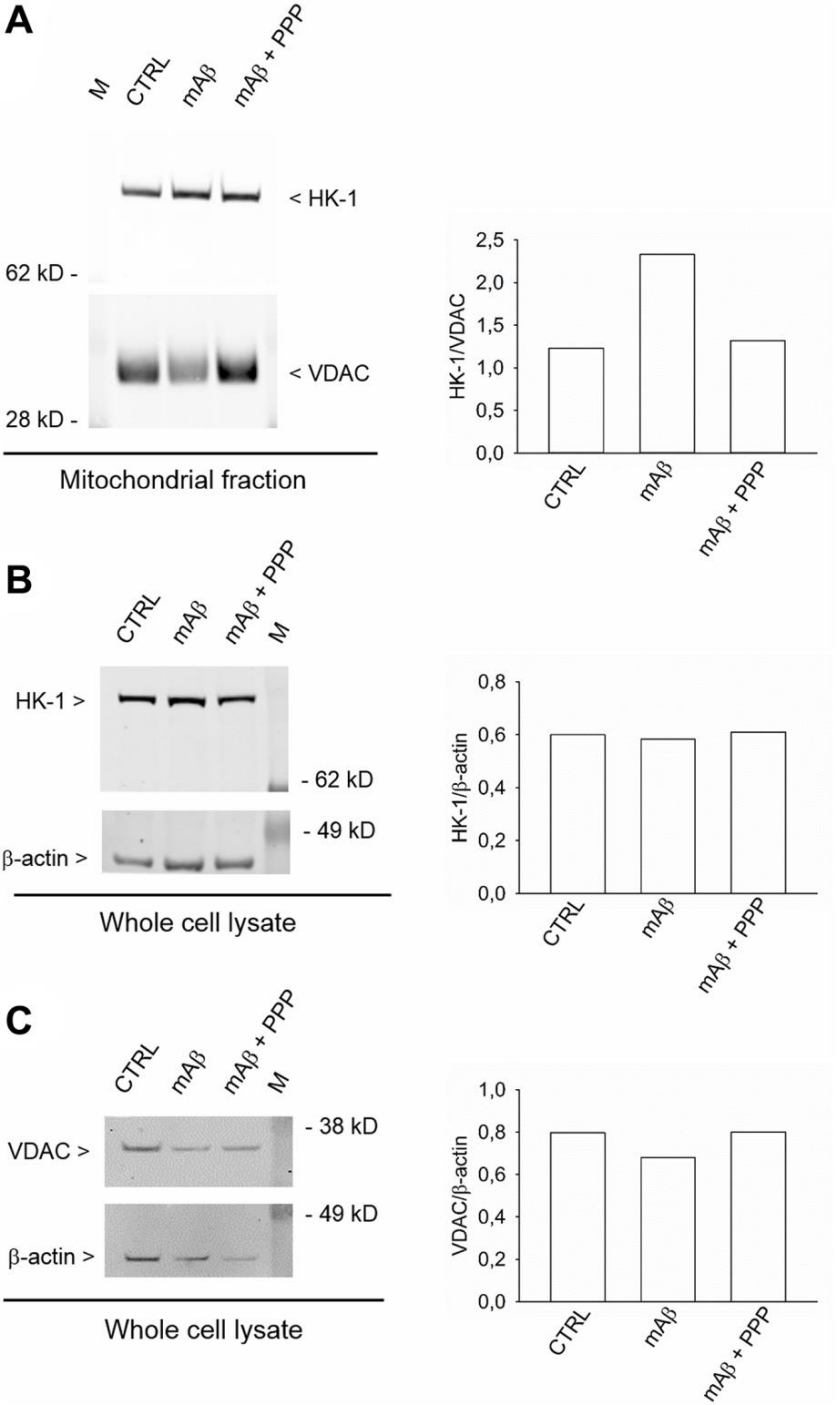
**Synthetic Aβ_42_ monomers enhanced the mitochondrial abundance of HK-1 without altering the total protein content.** In (**A**), the western blot analysis of HK-1 in the mitochondrial fraction of neurons that, following glucose deprivation and replenishing, were exposed to Aβ_42_ monomers in the absence (mAβ, 100 nM for 40 min) and in the presence of 500 nM PPP (mAβ ± PPP). Densitometric values of HK-1, normalized on VDAC signals, are represented in the graph bars (right). In (**B** and **C**), the western blot analysis of HK-1 and VDAC, respectively, in the whole neuronal lysate. Densitometric values of HK-1 or VDAC, normalized on β-actin signals, are represented in the respective graph bars (right). The whole cell lysate and the mitochondrial fraction were derived from the same experiment, but proteins were loaded in different amounts/gel (15 μg in (**A**), 20 μg in (**B**) and 10 μg in (**C**)) to avoid the saturation of hybridization signals. The experiment was repeated twice with similar results. Hybridization signals were detected with the Odyssey infrared imaging system in their original green or red colors and automatically converted into greyscale. M = protein marker.

We investigated whether Aβ_42_ monomers were able to increase the fraction of mitochondria-bound HK-1 under conditions of forced-glucose uptake. Neurons were starved for 2 hr before returning to 3 mM glucose in the absence or in the presence of synthetic Aβ_42_ monomers (100 nM for 40 min). When required, the IGF-IR antagonist, PPP (500 nM), was added. Immunofluorescence analysis, by confocal microscopy, of neurons labeled for HK-1 (green) (Figure 5A, 5D, 5G) and the voltage-dependent anion channel (VDAC) (red) OMM protein (Figure 5B, 5E, 5H), showed that HK-1 and VDAC signals overlapped almost perfectly (average Manders’ co-localization coefficients for the green channel and the red channel were 0.893 and 0.903, respectively) under basal conditions (Figure 5C). Interestingly, neurite processes in cultures exposed to synthetic Aβ_42_ monomers often exhibited a co-localization of the two fluorescence signals (Figure 5F), which was mostly absent in the presence of PPP (Figure 5I) and in the controls (Figure 5C). This subtle difference paralleled the increase in the percentage of image volume co-localized, which was observed in cultures exposed to Aβ_42_ monomers (Figure 5J). Hence, Aβ_42_ monomers appeared to increase the mitochondrial localization of HK-1 in a manner dependent on IGFI-R activation. Western blot analysis confirmed that Aβ_42_ monomers enhanced the amount of HK-1 that co-fractioned with neuronal mitochondria, when HK-1 densitometry signals were normalized against VDAC as fractionation control (Figure 6A). As in the case of immunofluorescence analysis, this effect was prevented by the addition of PPP to Aβ_42_ monomers (Figure 6A). The evidence that Aβ_42_ monomers did not really alter the total amounts of neuronal HK-1 (Figure 6B) and VDAC (Figure 6C), when the respective densitometry signals were normalized against β-actin as loading control, confirmed that the increase in the mitochondrial abundance of HK-1 was due to the enhanced association of the enzyme with the OMM.

**Figure 7:**
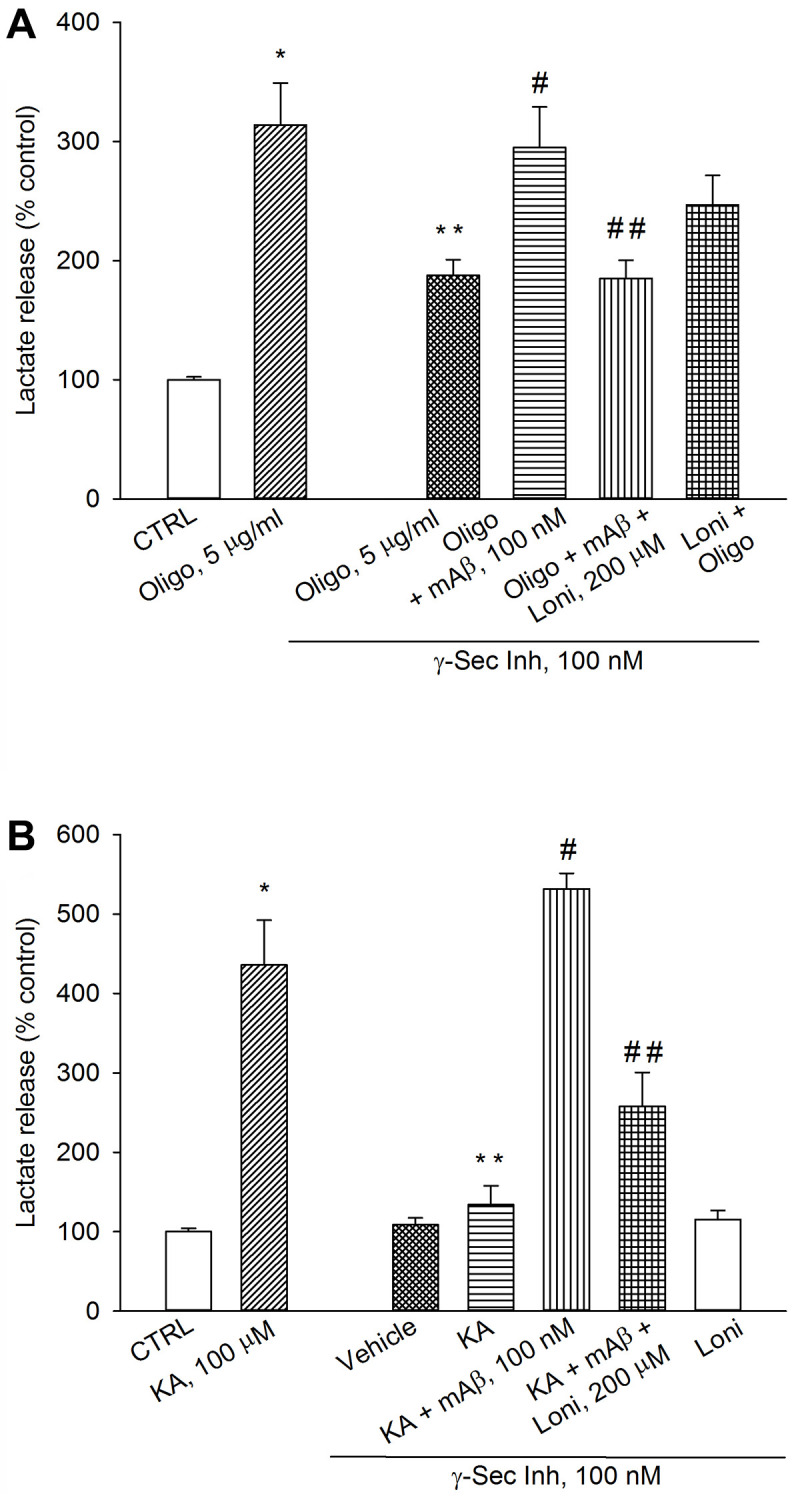
**Inhibition of mitochondria-bound HK-1 by lonidamine prevented lactate production due to Aβ release.** Neurons were glucose-starved for 2 hours before returning to 3 mM glucose. γ-Secretase inhibitor IX (γ-Sec Inh, 100 nM) reduced lactate release both in oligomycin-treated neurons (Oligo, 5 μg/ml for 1 hr) (**A**) and kainate-treated neurons (KA, 100 μM for 40 min) (**B**). The addition of synthetic Aβ_42_ monomers (mAβ, 100 nM) prevented the reduction of lactate release, induced by γ-Sec Inh, both in (**A** and **B**). Lonidamine (200 μM) reduced the rescuing effect of exogenous Aβ_42_ monomers in both cases (**A** and **B**). Bars represent the means ± SEM of 4 determinations. In (**A**) *p* < 0.001 vs. ^*^control (CTRL) or ^**^Oligo in the absence of γ-Sec Inh, and *p* < 0.05 vs. ^#^Oligo + γ-Sec Inh or ^##^Oligo + γ-Sec Inh + mAβ. In (**B**) *p* < 0.001 vs. ^*^control (CTRL) or ^**^KA in the absence of γ-Sec Inh or ^#^KA + γ-Sec Inh, and *p* < 0.05 vs. ^##^KA + γ-Sec Inh + mAβ; one-way ANOVA with post hoc Fisher LSD multiple comparison method.

To determine whether mitochondria-bound HK-1 was needed for Aβ-mediated AG under metabolic stresses, we used lonidamine, an inhibitor of mitochondria-bound HK [23]. As before, a pre-treatment with γ-secretase inhibitor IX reduced lactate release both in oligomycin-treated neurons (Figure 7A) and kainate-excited neurons (Figure 7B), which was rescued by adding synthetic Aβ_42_ monomers (Figure 7A, 7B). The addition of lonidamine (120 μM) prevented the rescuing effects of exogenous Aβ_42_ monomers in both cases (Figure 7A, 7B), indicating that Aβ_42_ monomers used mitochondrial HK-1 to favor lactate production and release.

Although Aβ42 monomers per se were able to promote the mitochondrial localization of HK-1 in response to glucose uptake (Figures 5F, 5J, 6A), they were not able to promote AG in the absence of oligomycin or kainate (Figure 4B). This evidence suggested that other key components facilitated Aβ-mediated lactate release under metabolic stresses.

**Figure 8:**
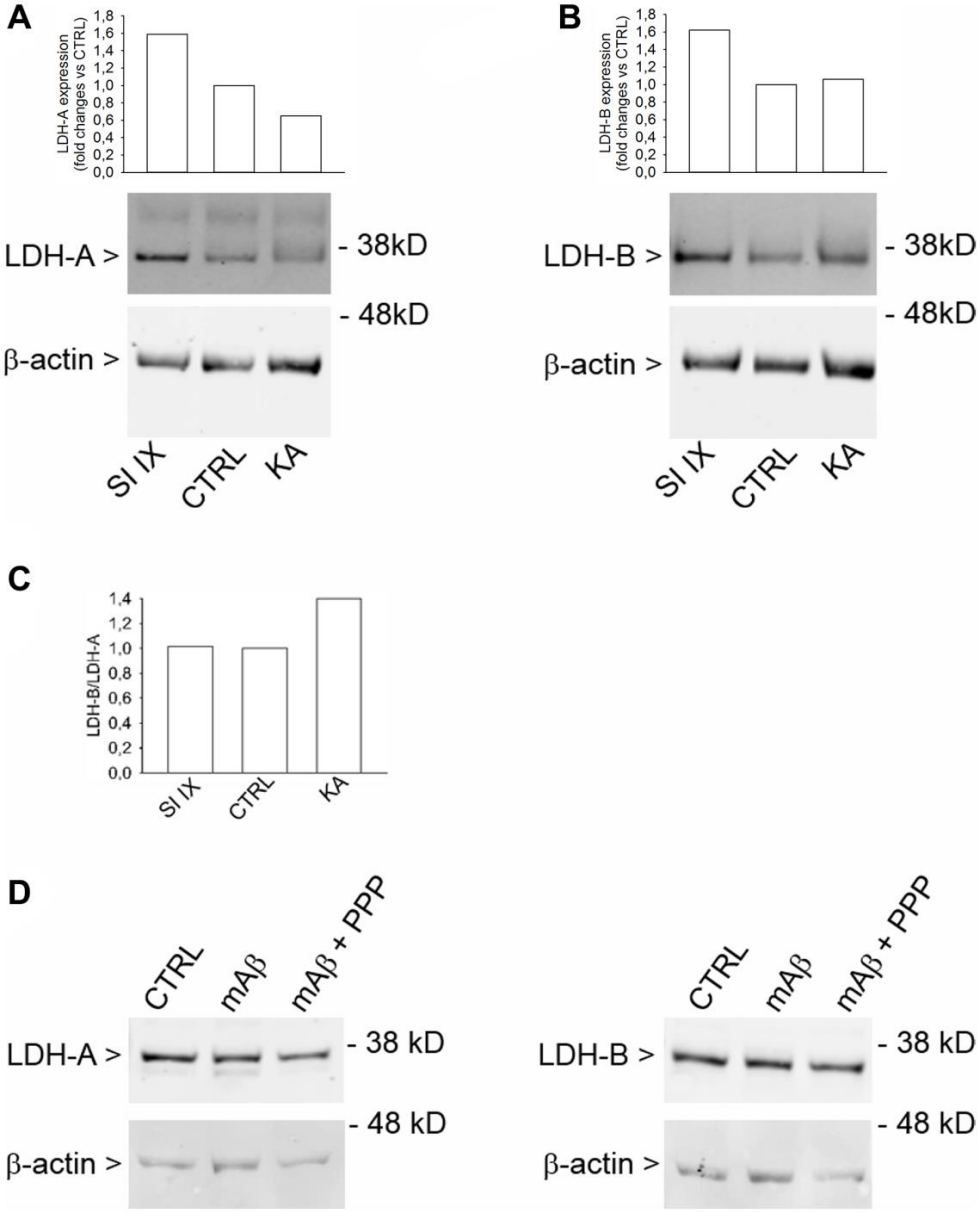
**Increased LDH-B/LDH-A expression ratio in neurons challenged with kainate.** Western blot analysis of LDH-A (**A**) and LDH-B (**B**) in lysates obtained from neurons that were deprived from glucose for 2 hr before returning to 3 mM glucose. Kainate (KA, 100 μM for 40 min) reduced LDH-A (**A**) without affecting LDH-B expression (**B**). γ-Secretase inhibitor IX (SI IX), 100 nM during glucose deprivation and for 40 min following glucose re-addition, increased both LDH-A (**A**) and LDH-B (**B**) expression. In (**A** and **B**), graph bars represent fold changes of LDH-A and LDH-B over the respective control (CTRL). Densitometry signals were normalized on β-actin. In (**C**), graph bars represent the ratio between LDH-B and LDH-A values as expressed in (**B** and **A**), respectively. The experiment was repeated twice with similar results. Hybridization signals were detected with the Odyssey infrared imaging system in their original green or red colors and automatically converted into greyscale. In (**D**), western blot images of LDH-A and LDH-B in lysates from neurons that, following glucose deprivation and replenishing, were exposed to Aβ_42_ monomers in the absence (mAβ, 100 nM for 40 min) and in the presence of 500 nM PPP (mAβ + PPP). None of the treatments modified LDH-A or LDH-B expression.

Lactate dehydrogenase (LDH) is a key enzyme that catalyzes the reversible conversion of pyruvate to lactate. LDH is a tetramer assembled by association of two different subunits, LDH-A and LDH-B. Five LDH isoenzymes exist that differ in their proportions of LDH-A and LDH-B subunits. The ratio of the two subunits determines the activity of LDH [24]. LDH-A mainly reduces pyruvate to lactate under anaerobic conditions, whereas LDH-B catalyzes the interconversion of low concentrations of pyruvate and lactate, as present in aerobic tissues, because of its high affinity for both substrates [25]. Western blot analysis of cell lysates obtained from neurons that had been exposed to kainate, as described previously, showed an increased LDH-B/LDH-A ratio (Figure 8C), mainly due to the reduction of the LDH-A expression (Figure 8A). γ-Secretase inhibitor IX, which per se never increased lactate release, did not affect the LDH-B/LDH-A ratio (Figure 8C), although it increased both LDH-A (Figure 8A) and LDH-B (Figure 8B) expression. Hence, the LDH-B/LDH-A ratio rather than the total content of LDH seemed relevant for lactate production by neurons. Accordingly, Aβ_42_ monomers, which promoted the mitochondrial localization of HK-1 in response to glucose uptake but not lactate production, did not affect the expression of either LDH-A and LDH-B (Figure 8D). Hence, the increased LDH-B/LDH-A ratio could act in tandem with the mitochondrial HK-1 to allow the Aβ-mediated lactate release under metabolic stresses.

## DISCUSSION

Energy metabolism in AD brain has been given increasing attention, mainly because regional metabolic deficits antedate the clinical onset of AD [26–28] and show no match with the areas of structural atrophy that are usually observed in early-stage AD [29]. Therefore, the unresolved question is the correlation between glucose hypometabolism, cognitive functions and AD neuropathology (i.e., amyloid plaques and neurofibrillary tangles). Based on the evidence that Aβ monomers are able to enhance glucose uptake in cultured neurons by activating IGF-IRs and promoting the membrane translocation of the Glut3 glucose transporter [15], we speculated that a reduced neuronal secretion of Aβ occurring with aging [30] or a loss of Aβ monomers due to the self-oligomerization process, could be related to the impairment of brain glucose uptake antedating the clinical onset of AD [31]. In this sense, was meaningful to us the evidence that a defective IGF-IR signaling was reported in AD brain tissue even in the absence of obvious plaque pathology [32].

Targeted investigations into the relationship among changes in brain energy metabolism, normal aging and AD have led to the conclusion that age affects brain metabolism independently of AD [1] and that age-related metabolic reductions largely overlay the DMN and concern specifically AG (i.e., the ability of nervous cells to quickly process glucose to the high energy intermediate, lactate) [1]. Because the DMN is prone to developing AD [3, 33], a loss of AG could be both a change related to aging and a factor of disease susceptibility. With this premise, and assuming that several factor can influence the availability of Aβ monomers, we intended to investigate the hypothesis that monomers were required to sustain neuronal AG via IGF-IR activation.

*In vitro*, both astrocytes [34] and neurons [12, 35] are able to increase glycolysis and secrete lactate after stimulation, and both cell types are capable of oxidizing the lactate that is provided to them exogenously [36–37]. Since we were interested in studying neuronal AG as a direct response of neurons to physiological forms of Aβ, we performed all experiments in mature cultures of pure cortical neurons to avoid potential confounds arising from the presence of glia cells. Moreover, since micronutrients are known to influence neuronal metabolism even in acute [38], we decided to perform short-term experiments in aCSF. Cultured neurons adapt quickly to metabolic challenges and recover their metabolic abilities following a non-lethal time of glucose deprivation [39]. We found that neuronal survival, after glucose deprivation and re-addition, did not entirely relied on OP or glucose metabolism through the PP shunt, suggesting a pro-survival role for AG. Accordingly, in response to the inhibition of OP by oligomycin, neurons largely increased their basal release of lactate and kept most of their ATP levels, which were both prevented by blocking the endogenous Aβ tone and re-established by the exogenous addition of synthetic Aβ monomers. Blockade of the endogenous Aβ tone was able to prevent AG even when stimulated by a kainate pulse, but never affected the basal release of lactate in the absence of a metabolic stressor (i.e., oligomycin or kainate). Similarly, Aβ monomers per se did not promote neuronal lactate release, suggesting that Aβ was physiologically required to sustain forced but not basal AG. In searching for the molecular tools necessary for Aβ monomers to sustain forced AG, we initially investigated the role of AMPK, an enzyme that has been shown to maintain cell energy levels during synaptic activation [40] or following OP suppression [19]. Surprisingly, the AMPK inhibitor, Compound C, did not prevent lactate release due to Aβ during OP blockade or kainate stimulation, suggesting that AMPK was not involved.

Based on these finding, we thought of investigating a molecular determinant intrinsic to the signaling pathway activated by Aβ, on the one hand, and a permissive component for Aβ-induced lactate release under conditions of metabolic stress, on the other. With respect to the first point, we focused on HK-1, which is able to translocate from the cytosol to the OMM in response to the activation of the PI-3K/AKT pathway [20] (i.e., the signaling pathway triggered by Aβ monomers [14]) and, as mitochondria-bound HK-1, is found in cells with a high rate of AG [21]. As assessed by confocal analysis, HK-1 was mostly mitochondrial under conditions of glucose uptake, and Aβ monomers, through the activation of IGF-IRs, appeared to increase further the mitochondrial localization of HK-1 in correspondence with neuronal processes. Although the relevance of mitochondria-bound HK-1 at neuritic level remains partly unclear, the use of lonidamine, an inhibitor of mitochondria-bound HK [23], demonstrated that Aβ monomers used mitochondrial HK-1 to support lactate release during OP blockade or kainate stimulation. Regarding the second aspect, namely the search for a key component facilitating Aβ-mediated lactate release under metabolic stresses, we focused on LDH. LDH catalyzes the bidirectional conversion of pyruvate and lactate and the direction of conversion, whether from pyruvate to lactate or vice versa, would appear to depend on the relative proportion of LDH-A and LDH-B type subunits that make up the enzyme [24]. We found that a 40 min pulse with kainate reduced neuronal LDH-A content and, consequently, the LDH-B/LDH-A ratio increased. This result disagrees with the old statement that LDH-A favors lactate production, while the LDH-B isoform favors pyruvate production [41]. Specifically, it has been suggested that an increased LDH-A/LDH-B ratio causes high brain lactate levels in response to a reduced mitochondrial oxidative capacity in a mouse model of advanced aging [42]. In APP/PS1 mice, an increased ratio of neuronal LDH-A/LDH-B has been proposed to occur as a reaction of neurons to a lactate deficit deriving from a reduced lactate transport from astrocytes to neurons [43]. Always in APP/PS1 mice, LDH-A is primarily expressed in neurons and astrocytes surrounding amyloid plaques, and is associated with high levels of lactate in the hippocampal interstitial fluid [44]. On the contrary, LDH-A has been found to be decreased in the cerebral cortex of aged mice, where LDH-B remains unchanged and lactate levels rise [45]. Overall, the role of LDH isoforms in lactate production versus utilization, as well as the relevance of total LDH content regardless of isoform patterns are not fully elucidated [46]. Our evidence that a kainate pulse increased both the LDH-B/LDH-A ratio and the release of lactate is in line with the suggestion that LDH-B can catalyze efficiently the conversion of low concentrations of pyruvate to lactate under normoxic conditions [25] and when the metabolic flux is positive (i.e., when pyruvate is supplied and lactate is released) [47]. At this stage, we can only hypothesize that the increase in the LDH-B/LDH-A ratio, induced by kainate, was permissive for Aβ-induced lactate release. It remains to be determined what would be the ultimate effects of the released lactate, which can be captured by neighboring neurons as metabolic fuel [12], is potentially able to modulate neuronal firing through membrane receptors [48], and can even regulate the expression of plasticity genes [49]. In the specific case of AD, lactate production has been seen as a transient compensation mechanism to maximize energy metabolism in the brain [44] and mitigate the toxic effects of Aβ aggregates by counteracting mitochondrial oxygen consumption and associated ROS production [10, 50]. Our data suggest that, through Aβ release, stimulated neurons coordinate glucose uptake with AG and, possibly, become lactate exporters. Several reports indicate that factors enhancing glucose uptake and glycolytic flux (e.g., Wnt3a) [51–52] or downregulating mitochondrial OP (e.g., soluble APP) [53] could be beneficial in AD. Hence, further studies are needed to understand the molecular mechanisms responsible for metabolic disturbances in early AD and enable new approaches to sustain the DMN efficiency.

## METHODS

### Primary neuronal cultures: preparation and treatments

Animal care and experimentation were in accordance with national and institutional guidelines. Cultures of pure cortical neurons were obtained from rats at embryonic day 15 and grown as described previously [14, 18]. Cortical cells were plated onto 35 mm dishes or glass bottom culture dishes pre-coated with 0.1 mg mL^−1^ poly-D-lysine and incubated at 37°C with 5% CO_2_ in a humidified atmosphere. Cytosine arabinoside (1-β-D-arabinofuranosylcytosine, Ara-C) (5 μM) was added to the cultures 18 h after plating to avoid the proliferation of non-neuronal elements and was kept for 3 days before partial medium replacement. Experiments were performed in mature culture at 13–17 days *in vitro* (DIV).

All experiments were performed in artificial excitable CSF (3.5 mM KCl, 126 mM NaCl, 1.25 mM NaH2PO4, 0.5 mM MgSO4, 1 mM CaCl2, 26 mM NaHCO3) containing 10 μM glycine and, when required, 3 mM glucose. To block Aβ release, 100 nM γ-secretase inhibitor IX was added to the artificial excitable CSF 2 hr before the experiments.

### Propidium iodide (PI) staining

For PI staining of dead neurons, culture dishes were washed once with PBS and exposed to the PI working solution (5 μg/ml) for 3 min. Then, dishes were returned to PBS and visualized by fluorescent microscopy. PI-positive neurons were scored from three random fields/dish.

### Glucose, lactate and ATP assays

Glucose content in the culture buffer was measured by Cayman’s Glucose Colorimetric Assay Kit. Lactate release in the culture buffer was quantified by Sigma-Aldrich Lactate Assay Kit (colorimetric detection). ATP content in neuronal lysates was measured by Rat ATP Elisa KIT (Creative Diagnostics). In all cases, following the technical instructions, absorbance was read by a spectrophotometric multiwell plate reader.

### Peptide monomers preparation

Aβ_1–42_ (HFIP-treated) was purchased from Bachem Distribution Services GmbH, Germany, dissolved at a 5 mm concentration in anhydrous dimethyl sulfoxide (DMSO) and stored at −20°C. At the time of its use, a solution of 100 μm Aβ in ice-cold DMEM F-12 was prepared and allowed to oligomerize overnight at 4°C according to our previously described method [14]. Monomers were isolated from the peptide suspension, containing both monomers and oligomers, by filtration through 10 kD cutoff filters as previously described [14].

### Confocal analysis

For confocal analysis, neurons were grown on glass bottom dishes (WillCo-dish^®^, Willco Wells, B.V., Amsterdam, The Netherlands). After the experiments, neurons were fixed in 2% paraformaldehyde and permeabilized using 0.1% Triton X-100. Unspecific binding was blocked by 30 min of incubation in 4% bovine serum albumin (BSA) in 0.1% Triton X-100-PBS. HK-1 was detected by incubating neurons for 2 hr with rabbit anti-HK-1 antibody (1:100, Abcam 150423). After PBS washing, neurons were exposed for 45 min to the anti-rabbit Alexa Fluor 488 antibody (1:500, Thermofisher). Cultures were blocked again with 4% BSA before second staining with rabbit anti-VDAC antibody (1:1000, Abcam 154856) for 2 hr, followed by 45 min exposure to the anti-rabbit Alexa Fluor 546 antibody (1:300, Thermofisher). Confocal images were acquired with an Olympus FV1000 confocal microscope, using two laser lines (Argon 488 nm and HeNe 543 nm) and two detection channels (500–530 nm and 550–600 nm) for the green and red false colour channels, employed to measure the brightness of HK-1 and VDAC, respectively. The detector gain was fixed at a constant value, with spectral filtering systems active, and images were collected, in sequential mode, randomly all through the area of the glass bottom dish by using an oil immersion objective (60xO PLAPO). The image deconvolution analysis was carried out using Huygens Essential software (by Scientific Volume Imaging B.V., The Netherlands). The co-localization analysis was performed by freely available ImageJ software.

### Western blot analysis

Western blotting analysis of LDH-A and LDH-B was performed with 20 μg of total proteins and samples were loaded onto 10% bis-Tris Plus gel (Bolt, Invitrogen). Western blotting analysis of HK-1 was performed with 20 μg of total proteins or 15 μg of mitochondrial proteins loaded onto 8% bis-Tris Plus gel. The mitochondrial fraction was obtained according to the protocol described by Schindler and Foley [54]. Western blotting analysis of VDAC was performed with 10 μg of total proteins loaded onto 4–12% bis-Tris Plus gel or 15 μg of mitochondrial proteins loaded onto 8% bis-Tris Plus gel. After separation, proteins were transferred onto a nitrocellulose membrane (Hybond ECL, Amersham Italia) using a transblot semi-dry transfer cell. Membranes were incubated over night at 4°C with the following primary antibodies: rabbit anti-LDH-A (1:250, MyBioSource 355106), rabbit anti-LDH-B (1:500, MyBioSource 9434882), rabbit anti-HK-1 antibody (1:10,000, Abcam 150423), rabbit anti-VDAC antibody (1:5,000, Abcam 154856), and mouse anti-ß-actin (1:1,500, Sigma Aldrich A4700). For the detection of hybridization signals, membranes were incubated with secondary goat anti-rabbit labeled with IRDye 800 (1:35,000 Li-COR Biosciences) and goat anti-mouse labeled with IRDye 680 (1:30,000 Li-COR Biosciences) for 45 min at RT. Signals were detected with the Odyssey Infrared Imaging System (LI-COR Biosciences).

### Statistical analysis

Quantitative data were expressed as the mean ± standard error (SEM). *P* values were calculated with analysis of variance (ANOVA), followed by post hoc Fisher LSD multiple comparison method or post hoc Holm-Sidak multiple comparisons vs. control group. Analysis was carried out using SigmaPlot 12.5.
